# New Applications to Ulcerated Surfaces

**Published:** 1870-04

**Authors:** 


New Application to Ulcerated Surfaces.—A solution of xyloidine and tannin in ether is praised by Dr. Richardson, of London, as an application to uterine ulcerations—so called. Dr. Richardson says this combination rapidly cicatrizes ulcerated or raw surfaces, and that it acts as an antiseptic by reason of the tannin it contains.

---

In Dr. Richardson's lecture, delivered on Tuesday last, he showed that hydrate of chloral produces sleep when administered as an enema as well as when given by the mouth or introduced subcutaneously. He also showed that butyl alcohol and chloride of amyl, when injected under the skin, produce a deep sleep similar to that from chloral.—*Medical Times and Gazette.*

